# *Mycobacterium tuberculosis* Acetyltransferase Suppresses Oxidative Stress by Inducing Peroxisome Formation in Macrophages

**DOI:** 10.3390/ijms23052584

**Published:** 2022-02-26

**Authors:** Ananyaashree Behera, Preeti Jain, Geetanjali Ganguli, Mainak Biswas, Avinash Padhi, Kali Prasad Pattanaik, Barsa Nayak, Süleyman Ergün, Kristine Hagens, Natalja Redinger, Mohd Saqib, Bibhuti B. Mishra, Ulrich E. Schaible, Srikanth Karnati, Avinash Sonawane

**Affiliations:** 1 School of Biotechnology, KIIT Deemed to be University, Bhubaneswar 751024, India; ananyaashree@gmail.com (A.B.); g220dec@gmail.com (G.G.); mainakbiswas.scc@gmail.com (M.B.); avinash.padhi134@gmail.com (A.P.); kaliprasad25@gmail.com (K.P.P.); 2 National Institute of Immunology, New Delhi 110067, India; jain.preeti35@nii.ac.in; 3 Department of Biosciences and Biomedical Engineering, Indian Institute of Technology Indore, Indore 453552, India; barshanayak50@gmail.com; 4 Institute of Anatomy and Cell Biology, Julius-Maximilians-University Würzburg, 97070 Würzburg, Germany; sueleyman.erguen@uni-wuerzburg.de; 5 Department of Cellular Microbiology, Program Area Infections, Research Center Borstel-Leibniz Lung Center, 23845 Borstel, Germany; khagens@fz-borstel.de (K.H.); nredinger@fz-borstel.de (N.R.); uschaible@fz-borstel.de (U.E.S.); 6 Department of Immunology and Microbial Disease, Albany Medical College, NY 12208, USA; saqibm@amc.edu (M.S.); mishrab@amc.edu (B.B.M.)

**Keywords:** peroxisome, *Mycobacterium tuberculosis*, Rv3034c, acetyltransferase, macrophages, oxidative stress

## Abstract

*Mycobacterium tuberculosis* (*Mtb*) inhibits host oxidative stress responses facilitating its survival in macrophages; however, the underlying molecular mechanisms are poorly understood. Here, we identified a *Mtb* acetyltransferase (Rv3034c) as a novel counter actor of macrophage oxidative stress responses by inducing peroxisome formation. An inducible *Rv3034c* deletion mutant of *Mtb* failed to induce peroxisome biogenesis, expression of the peroxisomal β-oxidation pathway intermediates (ACOX1, ACAA1, MFP2) in macrophages, resulting in reduced intracellular survival compared to the parental strain. This reduced virulence phenotype was rescued by repletion of *Rv3034c*. Peroxisome induction depended on the interaction between Rv3034c and the macrophage mannose receptor (MR). Interaction between Rv3034c and MR induced expression of the peroxisomal biogenesis proteins PEX5p, PEX13p, PEX14p, PEX11β, PEX19p, the peroxisomal membrane lipid transporter ABCD3, and catalase. Expression of PEX14p and ABCD3 was also enhanced in lungs from *Mtb* aerosol-infected mice. This is the first report that peroxisome-mediated control of ROS balance is essential for innate immune responses to *Mtb* but can be counteracted by the mycobacterial acetyltransferase *Rv3034c*. Thus, peroxisomes represent interesting targets for host-directed therapeutics to tuberculosis.

## 1. Introduction

*Mycobacterium tuberculosis* (*Mtb*), the most infectious pathogen, is accountable for significant morbidity and mortality worldwide. Every year, approximately 10 million people are infected, and 1.4 million people die globally due to tuberculosis (TB) [1,2]. The emergence of multidrug-resistant (MDR) and extensively drug-resistant (XDR) *Mtb* strains are posing a huge challenge on the control of TB [3]. Following inhalation, *Mtb* is phagocytosed by alveolar macrophages, which are equipped with various antibacterial effector mechanisms to eliminate the intracellular pathogens, such as the production of reactive oxygen intermediates (ROI), reactive nitrogen intermediates (RNI), and proinflammatory cytokines, and the induction of autophagy. However, *Mtb* has evolved diverse evasion strategies to subvert the immune and metabolic responses, thus avoiding being killed by the hostile intracellular microenvironment of the host cells [4]. Studies have revealed distinct phenotypic heterogeneity of macrophages associated with functional plasticity, allowing differential responses and changes of their intracellular microenvironment depending on the *Mtb* strain encountered [5].

Numerous studies have established mitochondria as a prime site of immune and inflammatory responses against bacterial infections [6]. However, recent evidence suggests that peroxisomes can also be crucial for the regulation of key immune responses during infections [7]. Peroxisomes are dynamic and ubiquitous organelles separated by a single lipid membrane from the cytoplasm. They have the capability to adapt to morphological and functional changes of a cell responding to environmental stimuli. Peroxisomes play a crucial role in the regulation of reactive oxygen species (ROS) production and the catabolism of long-chain fatty acids [8,9]. These functions of peroxisomes are well studied in eukaryotes, plants, and fungi; however, their role in bacterial infection, specifically during mycobacterial infection, is poorly understood [10,11,12]. A study by Karnati et al. demonstrated the presence of peroxisomes in all cell types of the human lung, with heterogeneity in their structure, numerical abundance, and enzyme composition depending on the metabolic needs of the equivalent cell types [13]. Peroxisomes contain an array of oxidases that generate hydrogen peroxide (H_2_O_2_), such as flavin oxidase, urate oxidase (UoX), and acetyl CoA oxidase. Intracellular redox homeostasis is maintained by the degradation of toxic superoxide radicals into simpler forms by catalase and other antioxidative enzymes present inside the peroxisomes [11,14]. Furthermore, the abundance of β-oxidation enzymes and lipid transporter ABCD3 in lung peroxisomes indicates their importance in lipid metabolism and regulation of lipid signaling in the lung [13].

It is well established that *Mtb* suppresses oxidative stress responses in macrophages to promote its intracellular survival [15]. Our group demonstrated that *Mtb* modulates pexophagy mechanisms to maintain cellular peroxisome homeostasis essential to sustain intracellular redox balance [16]. As peroxisomes are known to control inflammation and maintenance of oxidative balance, targeting peroxisomes by modulating their function to maintain intracellular redox balance could be effective in the control of bacterial infection [17]. Here, we report for the first time that *Mtb* modulates peroxisomes to support its survival in macrophages. Further, we identify an *Mtb* acetyltransferase, encoded by *Rv3034c*, which is responsible for the induction of peroxisome biogenesis and the β-fatty acid oxidation pathway via interaction with macrophage MR. By constructing a conditional *Mtb Rv3034c* mutant, we demonstrate its potential to suppress host oxidative responses by inducing peroxisome biogenesis and peroxisomal β-oxidation pathways to scavenge ROS and thereby create a favorable niche for the persistence of mycobacteria in macrophages.

## 2. Results

### 2.1. M. tuberculosis Infection Induces the Expression of Proteins Involved in Peroxisome Biogenesis in Mice Lungs and Bone-Marrow-Derived Macrophages

**Figure 1:**
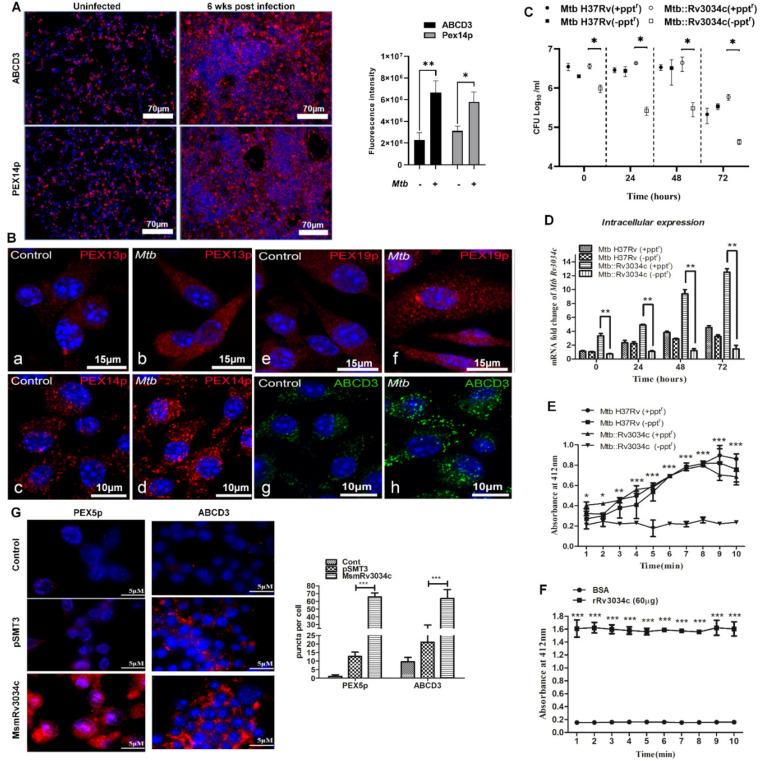
Determination of peroxisome formation in *Mtb*-infected mice and BMDM and determination of survival, expression, and acetyltransferase activity of *Rv3034c*. (**A**) Immunofluorescence analysis to determine the expression of peroxisome markers (ABCD3 and PEX14p) in control and *Mtb*-infected C57BL/6 mice (*n* = 5, male). Fluorescence intensity quantification has been represented in bar graph form. (**B**) Determination of different peroxisome-related markers in control and *Mtb*-infected BMDM using confocal microscopy. (**C**) Determination of intracellular survival of *Mtb* H37Rv and *MtbRv3034c* conditional mutants in mouse peritoneal macrophages. Mycobacterial strains grown in the presence and absence of pristinamycin are designated as “+ppt^r^” and “−ppt^r”^, respectively. Mouse peritoneal macrophages were infected with *Mtb* H37Rv (−ppt^r^), *Mtb* H37Rv (+ppt^r^), *Mtb*::Rv3034c (−ppt^r^), and *Mtb*::Rv3034c (+ppt^r^). The cells were lysed 0, 24, 48, and 72 h post-infection and the bacterial survival was determined by CFU assay. (**D**) qRT-PCR analysis to determine the intracellular expression of *Rv3034c*. Total RNA was isolated from *Mtb* H37Rv-(−ppt^r^)-, *Mtb*-H37Rv-(+ppt^r^)-, *Mtb*::Rv3034c-(−ppt^r^)-, and *Mtb*::Rv3034c-(+ppt^r^)-infected THP-1 cells. (E and F) Acetyltransferase activity was determined in protein lysates prepared from (**E**) *Mtb* H37Rv (±ppt^r^) and *Mtb*::Rv3034c conditional mutants grown in absence (−ppt^r^) and presence (+ppt^r^) of pristinamycin in 7H9 medium for 24 h, and (**F**) purified rRv3034c protein. BSA was used as a negative control. 60 µg of protein lysates were added to the substrate and the absorbance was measured at 412 nm. The absorbance values plotted were obtained after deduction from the absorbance value of the control sample. (**G**) Immunofluorescence studies were performed to determine the expression of PEX5p and ABCD3 in *Msm*-pSMT3- and *Msm-Rv3034c*-infected macrophages after 24 h. The puncta per cell were represented in bar graph form. Experiments were performed in duplicates. Statistical significance was performed with one-way ANOVA. For acetyltransferase activity, statistical significance was performed with two-way ANOVA Bonferroni post-tests. Data represent mean ± SD; * for *p* ≤ 0.05, ** for *p* ≤ 0.01, and *** for *p* ≤ 0.001.

We previously observed that modulation of pexophagy mechanisms is necessary for maintenance of peroxisomes during mycobacterial infection [16]. Here, we aimed to understand the molecular mechanisms of induction of peroxisome biogenesis during *Mtb* infection and its consequences for intracellular persistence of mycobacteria. To test this, we infected mice with *Mtb*H37Rv via the aerosol route and the expression of peroxisomal proteins in infected lungs was determined. Immunofluorescence imaging of lung tissue sections showed a significant increase in the expression of peroxisomal lipid transporter ABCD3 and marker protein for peroxisomes PEX14p, which are responsible for the transport of cargo proteins and enzymes in peroxisomes, as compared to uninfected lungs (*p* ≤ 0.01, *p* ≤ 0.05, Figure 1A). H&E staining showed larger areas of inflammatory infiltrates at the site of infection in *Mtb*-infected lung tissue (Figure S1(IA)). We next assessed the abundance of several peroxisomal proteins in *Mtb*-infected murine bone marrow-derived macrophages (BMDM). *Mtb*-infected cells had a higher abundance of several proteins such as ABCD3, PEX13p, PEX14p, and PEX19p, which are involved in peroxisome biogenesis, as compared to uninfected cells at 24 h post-infection(p.i.) (Figure 1(Ba–Bh)). These results suggest that *Mtb* is able to induce the expression of proteins involved in the biogenesis and transporters of peroxisomes.

### 2.2. Absence of M. tuberculosis Acetyltransferase Reduces Bacterial Growth in Macrophages

We have shown that *Mtb* acetyltransferase, encoded by *Rv3034c*, is responsible for the regulation of peroxisomes in macrophages [16]. Studies have shown that acetyltransferases play an important role in bacterial pathogenesis [18,19]. Therefore, we investigated whether *Mtb Rv3034c* supported the intracellular growth of bacteria in macrophages. For this, we constructed the *Mtb Rv3034c* conditional mutant under the control of the inducible pristinamycin promoter (Figure S1(IB)). Using this approach, the addition of pristinamycin would induce the expression of *Rv3034c* (*Mtb*::Rv3034c[+ppt^r^]), whereas the absence of pristinamycin would result in *Rv3034c* depletion (*Mtb*::Rv3034c[−ppt^r^]). A schematic representation of the construction of the *Mtb Rv3034c* conditional mutant is shown in Figure S1(IB). Genetic modification at the native locus was confirmed by PCR using ppt^r^ promoter-specific forward and *Rv3034c* gene-specific reverse primers (Table 1) (Figure S1(IC)). Specific bands of the correct sizes (1.05 kb and 750 bp) for the *Mtb Rv3034c* conditional mutant were amplified, suggesting a successful construction of *Mtb Rv3034c* conditional mutant. Infection of mice peritoneal macrophages showed decreased survival of *Mtb Rv3034c* conditional mutant in the absence of pristinamycin (*Mtb*::Rv3034c[−ppt^r^]) at all indicated time points compared with *Mtb*::Rv3034c (+ppt^r^), *Mtb* (−ppt^r^), and *Mtb* (+ppt^r^) strains (*p* ≤ 0.01, Figure 1C). Mycobacterial strains grown in the presence and absence of pristinamycin are designated as “+ppt^r^” and “−ppt^r”^, respectively. No significant differences in the growth patterns of *Mtb* (−ppt^r^), *Mtb* (+ppt^r^), *Mtb*::Rv3034c (−ppt^r^), and *Mtb*::Rv3034c (+ppt^r^) were observed (Figure S1(ID)), suggesting that expression or depletion of *Rv3034c* in *Mtb* did not affect the bacterial growth kinetics. Further, we confirmed the expression of *Rv3034c* in *Rv3034c* expressing *Mtb* strains. As expected, significantly high *Rv3034c* expression was observed in *Mtb*::Rv3034c (+ppt^r^) compared with *Mtb*::Rv3034c (−ppt^r^), *Mtb* (−ppt^r^), and *Mtb* (+ppt^r^) strains (*p* ≤ 0.01, Figure 1D).

Given the importance of Rv3034c in *Mtb* pathogenesis, we first characterized the Rv3034c protein. Our in silico analysis using TBpred, TMpred, EnsemgleGly, Net-N-Glyc, and Net-O-Glyc predicted *Mtb* Rv3034c as integral cell-membrane-associated and potentially O-linked glycosylated at Ser 186 of Rv3034c. The TMpred analysis showed two transmembrane regions (Figure S1(IE)) at 9–29 and 45–65 amino acids, respectively. Therefore, we used various lectins, which exhibit affinity toward different types of sugar moieties, to capture Rv3034c from purified *Mtb* cell walls. We could successfully capture Rv3034c protein using a ConA lectin, which specifically binds to mannose residues (Figure S1(IF)). Using LC-ESI mass spectrometry, we identified this Con-A lectin captured 33-kDa putatively mannosylated protein as acetyltransferase (Rv3034c), from purified *Mtb* cell walls (Figure S1(IF)). The experimental peak masses, corresponding theoretical masses, and sequence matches are summarized in Table 2. To further confirm that Rv3034c is indeed a glycoprotein, we first expressed and purified the full-length Rv3034c as His-tagged protein (Figure S1(IG)). A glycostaining assay using a periodic acid-Schiff base that oxidizes the sugar moieties to produce red/pink-colored bands showed strong glycoprotein-positive staining with purified Rv3034c protein (Figure S1(IH), lane 2) and different concentrations of snail glycoprotein (lanes 3 and 4), while no staining was observed in the negative control of soybean trypsin inhibitor (lane 5).

### 2.3. M. tuberculosis Rv3034c Encodes an Acetyltransferase Enzyme

Following the identification and characterization of Rv3034, we next confirmed that *Mtb* Rv3034c indeed encodes for an acetyltransferase by measuring its enzymatic activity using Elman’s reagent, which produces a yellow color following ionization of 2-nitro-5-thiobenzoate (TNB) to TNB^2^ [20]. We observed significantly higher acetyltransferase activity in the *Rv3034c*-expressing *Mtb* and *Mtb*::Rv3034c (+ppt^r^) strains compared with the *Rv3034c*-depleted *Mtb*::Rv3034c (−ppt^r^) strain (*p* ≤ 0.05; *p* ≤ 0.01, and *p* ≤ 0.001, Figure 1E). Furthermore, recombinant purified Rv3034c protein (rRv3034c) (60 µg) exhibited significantly higher acetyltransferase activity than BSA (*p* ≤ 0.001, Figure 1F). Additionally, heat-inactivation of purified rRv3034c protein reduced the acetyltransferase activity (Figure S1(II)). These results demonstrate that *Rv3034c* encodes for an acetyltransferase enzyme.

### 2.4. Recombinant M. smegmatis Expressing Rv3034c and Purified Rv3034c Protein Induced Peroxisome Biogenesis

To further confirm the role of Rv3034c in peroxisome biogenesis, we constructed a recombinant *M. smegmatis* strain (*Msm*Rv3034c) that ectopically expresses *Mtb Rv3034c*. Several studies, including ours, have used *Msm* as a surrogate model to study the function of the *Mtb* proteins and identify genetic loci implicated in pathogenesis [21,22,23,24]. Like *Mtb*, no significant differences in the growth patterns of *Msm* wild-type (WT), vector control *Msm* pSMT3, and recombinant *Msm Rv3034c* strains were observed (Figure S1(IIA)). A time-dependent increase in the transcript of *Rv3034c* was observed in the *Msm Rv3034c* strain in a macrophage infection (Figure S1(IIB)).

Immunofluorescence analysis showed a significant fivefold increase in the expression of peroxisomal cytosolic receptor PEX5p (*p* ≤ 0.001) and peroxisomal ABC transporter ABCD3 (*p* ≤ 0.001) in mouse macrophages infected with *Msm* Rv3034c when compared with *Msm* pSMT3 (*p* ≤ 0.001, Figure 1G). We then confirmed the role of Rv3034c in the induction of peroxisome biogenesis by determining the expression of peroxisome membrane biogenesis (PEX19p), peroxisome proliferation (PEX11β), PEX5p and ABCD3 in macrophages infected with *Msm Rv3034c* (Figure S1(IIC)) and treated with recombinant Rv3034c protein (Figure S1(IID)). Western blot analysis demonstrated a significant increase in abundance for these peroxisomal proteins in *Msm-Rv3034c*-infected and Rv3034c-protein-treated macrophages compared with *Msm pSMT3* and untreated macrophages, respectively. These results further confirm that *Mtb* Rv3034c induces peroxisome biogenesis in macrophages.

### 2.5. M. tuberculosis Rv3034c Induced Peroxisome Biogenesis Scavenges Reactive Oxygen Species Production in Macrophages

**Figure 2:**
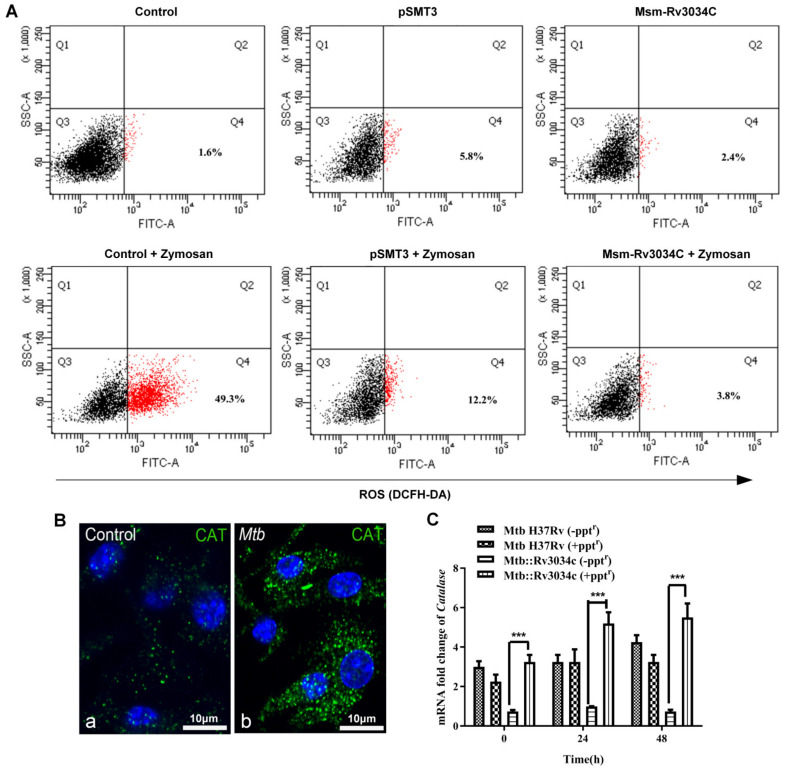
Determination of ROS production and expression of peroxisomal catalase in *Mtb*-infected BMDM and THP-1 cells. (**A**) ROS production was determined in *Msm*-pSMT3- and *Msm*-Rv3034c-infected and *Msm*-Rv3034c-plus-zymosan- (200 μg/mL) treated macrophages by DCFH-DA staining using flow cytometry. (**B**) Determination of peroxisomal catalase in control and *Mtb*-infected BMDM using confocal microscopy. (**C**) qRT-PCR of catalase was performed from total RNA isolated from THP-1 cells infected with *Mtb* H37Rv (−ppt^r^), *Mtb* H37Rv (+ppt^r^), *Mtb*::Rv3034c mutant (−ppt^r^), and *Mtb*::Rv3034c (+ppt^r^) after 24 h. The expression values were normalized with the *GAPDH* gene. Experiments were performed in duplicates. Statistical significance was performed with one-way ANOVA. Data represent mean ± SD; *** for *p* ≤ 0.001.

*Mtb* suppresses host oxidative stress responses and induces redox imbalance to avoid killing by the host cells [25]. As shown above (Figure 1C), depletion of *Rv3034c* reduced *Mtb* survival in macrophages, therefore, we asked whether *Mtb Rv3034c* modulates host oxidative stress responses to promote bacterial survival in macrophages. 2ʹ,7ʹ-Dichlorofluorescin diacetate (DCFH-DA) staining showed decreased ROS production in macrophages infected with *Msm* Rv3034c (2.4%) as compared to the ones infected with vector control pSMT3 (5.8%) (Figure 2A). Cells treated with zymosan were considered as a positive control for intracellular ROS production (Figure 2A).

Among several other peroxisomal enzymes, catalase and superoxide dismutase (SOD) play a key role in the maintenance of cellular redox homeostasis in host cells. Since Rv3034c was found to induce peroxisome biogenesis, we therefore assessed the synthesis of ROS scavenging enzymes in infected macrophages. Immunofluorescence analysis demonstrated higher peroxisomal catalase abundance in *Mtb*-infected BMDM (Figure 2B). qRT-PCR analysis also showed significantly higher levels of catalase in *Rv3034c*-expressing compared to *Rv3034c*-depleted *Mtb*-infected macrophages (*p* ≤ 0.001, Figure 2C). Furthermore, the expression of catalase was also significantly increased at transcriptional (*p* ≤ 0.05, Figure S2A) and translational (Figure S2B) levels in *Msm*-*Rv3034c*-infected macrophages. These results indicate that *Mtb Rv3034c* induces the expression of the peroxisomal antioxidative enzyme catalase as a putative scavenger of ROS in infected macrophages.

### 2.6. Rv3034c Induces the Peroxisomal β-Oxidation Pathway

**Figure 3:**
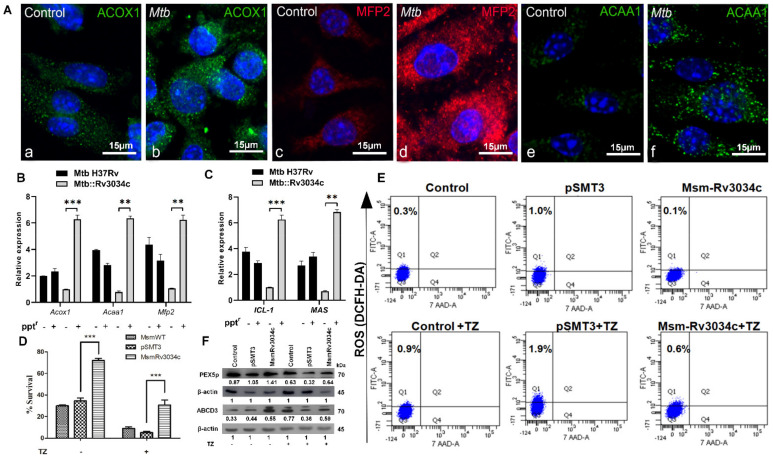
Analysis of expression of peroxisomal β-fatty acid oxidation and bacterial glyoxylate cycle intermediates in *Mtb*-infected BMDM and THP-1 cells and determination of *Rv3034c* survival, ROS production, and expression of peroxisomal proteins after treatment with peroxisomal β-oxidation inhibitor. (**A**) Determination of different peroxisomal β-fatty acid oxidation markers in control and *Mtb*-infected BMDM using confocal microscopy. (**B**,**C**) qRT-PCR analysis to determine the expression of peroxisomal β-fatty acid oxidation (**B**) and glyoxylate genes (**C**) from the total RNA isolated from THP-1 cells infected with *Mtb* H37Rv (−ppt^r^), *Mtb* H37Rv (+ppt^r^), *Mtb*::Rv3034c (−ppt^r^), and *Mtb*::Rv3034c (+ppt^r^). The expression values were normalized with *GAPDH* and *sigA* genes. (**D**) Intracellular survival of *Msm*WT, *Msm* pSMT3, and *Msm Rv3034c* strains in untreated and TZ-treated macrophages after 24 h. (**E**) Determination of ROS production by DCFH-DA staining in untreated (upper panel) and TZ-treated (lower panel) macrophages. Macrophages were infected with *Msm* pSMT3 and *Msm Rv3034c* for 24 h in presence of TZ. (**F**) Western blot analysis was performed to check the expression of peroxisome markers (PEX5p and ABCD3) in RAW264.7 macrophages infected with *Msm* pSMT3 and *Msm Rv3034c* in absence and presence of peroxisome inhibitor (TZ) after 24-h infection. Experiments were performed in duplicates. Statistical significance was performed with two-way ANOVA. Data represent mean ± SD; ** for *p*≤ 0.01 and *** for *p* ≤ 0.001.

As shown above, *Mtb Rv3034c* induces peroxisomes possibly to scavenge oxidative radicals to avoid killing by host phagocytes. It is well established that peroxisomes use oxidative radicals to perform β-fatty acid oxidation reactions to break down long-chain fatty acids present in the peroxisomes [26,27]. The products of peroxisomal β-oxidation (acetyl CoA and short-chain fatty acids) are metabolized to be used as nutrient sources. Therefore, we analyzed whether *Mtb Rv3034c* can also modulate the peroxisomal β-oxidation pathway to favor bacterial persistence in macrophages. For this, we measured the abundance of enzymes involved in the peroxisomal β-oxidation pathway in *Mtb*-infected macrophages. As shown in Figure 3A, immunofluorescence analysis revealed higher expression of acyl CoA oxidase 1 (ACOX1), multifunctional protein 2 (MFP2), and 3-ketoacyl-CoA thiolase (ACAA1) in *Mtb*-infected compared to non-infected macrophages. Similarly, qRT-PCR data showed an increase in the expression of these enzymes in the presence of *MtbRv3034c*; while in the absence of *Rv3034c* expression, levels of these enzymes were decreased (*p* ≤ 0.001 and *p* ≤ 0.01, Figure 3B).

Eukaryotic peroxisomal β-oxidation is directly linked to the bacterial glyoxylate cycle. A significant reduction in the expression of two bacterial glyoxylate enzymes, namely isocitrate lyase (*Icl1*) and malate synthase (*Mas*), was observed in *Mtb*::Rv3034c-(−ppt^r^)-infected cells compared with *Mtb*-(−ppt^r^)-, *Mtb*-(+ppt^r^)-, and *Mtb*::Rv3034c-(+ppt^r^)-infected cells (*p* ≤ 0.001 and *p* ≤ 0.01, Figure 3C). These results suggest that *MtbRv3034c* induces peroxisomal β-oxidation and the end products might be channeled by *Mtb* as a nutritional source into the glyoxylate pathway.

### 2.7. Inhibition of Peroxisomal β-Oxidation Decreases Bacterial Survival Due to Increase in the ROS Production

Previous studies have reported that peroxisomal β-oxidation is directly coupled to ROS production. This prompted us to assess whether inhibition of peroxisomal β-oxidation influences intracellular bacterial survival. For this, we pretreated macrophages with thioridazine hydrochloride (TZ, 10 µM), a selective inhibitor of peroxisomal β-oxidation [28], followed by infection with mycobacteria. Pre-treatment with TZ decreased intracellular survival of *Msm Rv3034c* by two-fold compared to untreated cells (*p* ≤ 0.001, Figure 3D). Treatment of infected macrophages with TZ also abrogated the *Rv3034c*-induced expression of peroxisomal β-oxidation enzymes *(Acox1, Mfp2* and ACAA1) at the transcriptional and translational levels (Figure S3A,B), as well as transcription of the bacterial glyoxylate cycle enzymes *(Icl1* and *Mas)* (Figure S3A).

DCFH staining showed that pretreatment with TZ relatively increased ROS production (0.6%) compared with untreated cells (0.1%) in the presence of *Rv3034c* (Figure 3E). Of note, the levels of ROS in *Msm*-*Rv3034c*-infected cells were still lower in the presence or absence of the inhibitor as compared to *Msm*-pSMT3-infected cells. Furthermore, exposure to TZ also decreased the expression of PEX5p and ABCD3 in the presence of *Mtb Rv3034c* as compared to untreated cells (Figure 3F). These data further corroborate *Mtb* Rv3034c as a counteractor of ROS production, which thereby induces peroxisome expression and promotes *Mtb* survival inside macrophages.

### 2.8. M. tuberculosis Rv3034c Induces Peroxisomes through the Macrophage Mannose Receptor (MR)

**Figure 4:**
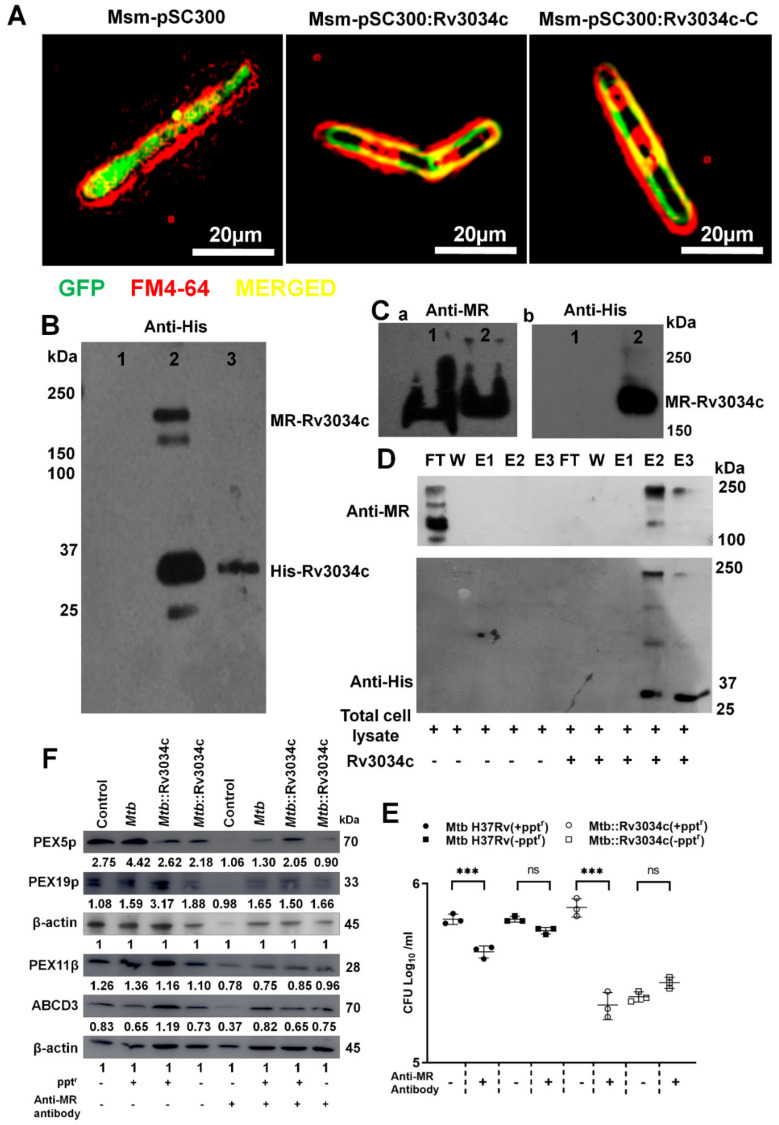
Cellular localization of Rv3034c and analysis of *Mtb* Rv3034c and mannose receptor interaction and expression of peroxisomal proteins in infected THP-1 cells. (**A**) Localization of Rv3034c in Msm-pSC300, Msm-pSC300:Rv3034c, and Msm-pSC300:Rv3034c-C strains using FM4-64 membrane staining dye by fluorescence microscopy. (**B**) Western blot analysis was performed to check the interaction of *Mtb* Rv3034c with mannose receptor (MR) using anti-His Tag antibody. Lane 1- whole cell protein (WCP) isolated from mouse macrophages (RAW264.7) expressing mannose receptor (MR), Lane 2- purified Rv3034c protein (containing His Tag) mixed with WCP isolated from RAW264.7 cells, and Lane 3- purified Rv3034c protein. (**C**) Western blot analysis to determine the interaction between Rv3034c and MR using MR antibody. Lane 1-whole cell protein isolated from RAW264.7 cells and Lane 2- purified Rv3034c protein (containing His Tag) mixed with WCP lysate of RAW264.7. The membrane was probed with anti-MR antibody (a) and anti-His tag antibody (b). (**D**) Ni-NTA affinity pull-down assay was performed to check the interaction of *Mtb* Rv3034c with MR using anti-His Tag antibody. First five lanes correspond to only WCP lysates isolated from RAW264.7 cells (expressing MR) and the last five lanes correspond to WCP lysates and purified Rv3034c-His protein (MR-Rv3034c-His protein) incubated with Ni-NTA beads. The eluted fractions were separated on 12% SDS-PAGE and Western blot was done using anti-MR and His-Tag antibodies. (**E**) Intracellular survival of *Mtb* H37Rv and *Mtb Rv3034c* conditional mutants in presence and absence of MR blocker in mouse peritoneal macrophages. Macrophages were infected with *Mtb* strains in presence and absence of pristinamycin and anti-MR antibody for 24 h. Intracellular bacterial survival was determined by CFU assay. (**F**) Determination of expression of peroxisomal proteins in presence and absence of MR blocker and different *Mtb*-strain-infected cells. THP-1 cells were infected with *Mtb* H37Rv, *Mtb*::Rv3034c (−ppt^r^), and *Mtb*::Rv3034c (+ppt^r^) in presence and absence of mannose receptor blocker. Protein lysates were prepared after 24 h of infection, and the expression of PEX11β, PEX19p, PEX5p, and ABCD3 was determined by Western blot analysis. Experiments were performed in duplicates. For *Mtb* survival, statistical analysis was performed with one-way ANOVA. Data represent mean ± SD; *** for *p* ≤ 0.001 and ns for non-significant.

Our glycoprotein staining assay and protein identification following ConA lectin chromatography analyses of purified *Mtb* cell walls suggested that Rv3034c could be a cell-wall-associated mannosylated glycoprotein (Figure S1(IF–IH). *Mtb* is known to affect host immune responses through interactions with Toll-like receptors (TLRs) and mannose receptors (MRs) present on the macrophage surface. Other *Mtb*-mannosylated glycolipoproteins, for example PstS-1, were shown to bind to MR [29]. We hypothesized that *Mtb* Rv3034c can modulate peroxisomes through interaction with MRs. For this, we first confirmed subcellular localization of Rv3034c by staining the membrane of *Msm*-pSC300, *Msm*-pSC300:Rv3034c, and *Msm*-pSC300:Rv3034c-C bacteria expressing a Rv3034c:GFP fusion protein with a membrane-specific FM4-64 dye. Intense colocalization of FM4-64 and Rv3034c-GFP was observed in the case of *Msm*-pSC300:Rv3034c (middle panel, Figure 4A). No such intense colocalization was observed in the *Msm*-pSC300 vector control strain, where a discrete green fluorescence was observed throughout the bacterial cells (left panel). *Msm*-pSC300:Rv3034c-Cter, which expresses the GFP-tagged Rv3034c C-terminus, coding for the acetyltransferase domain, also showed higher colocalization with FM4-64 compared with *Msm*-pSC300 bacteria (right panel, Figure 4A). This membrane colocalization of truncated Rv3034c is likely due to the presence of transmembrane domains in the C-terminus of Rv3034c.

Next, we confirmed whether Rv3034c regulates peroxisomes through interaction with MRs. For this we incubated purified His-tagged Rv3034c protein with whole-cell protein lysates isolated from macrophages expressing MR. The samples were separated by SDS-PAGE. Immuno-blotting with an anti-His antibody showed two prominent bands of ~220- and ~33-kDa, corresponding to the MR-Rv3034c complex and Rv3034c protein, respectively (Figure 4B, Lane 2). No bands were observed for the control of whole-cell protein lysate from macrophage alone (Lane 1), and only one band was observed for the control of His-tagged Rv3034c protein alone (Lane 3). These results provide evidence that Rv3034c interacts with MR. A Western blot of whole-cell protein lysate incubated with purified His-tagged Rv3034c protein and whole-cell protein lysate alone, after separation by native-PAGE, was probed with anti-MR (Figure 4(Ca)) and anti-His antibodies (Figure 4(Cb)). An upward shift was observed for the band in Figure 4(Ca), Lane 2, (whole-cell protein lysate incubated with purified His-tagged Rv3034c), compared to the band for the whole-cell protein lysate alone (Figure 4(Ca), Lane 1), indicating an interaction between MR and Rv3034c. This was further validated by the presence of a band corresponding to the whole-cell protein lysate incubated with purified His-tagged Rv3034c protein (Figure 4(Cb), Lane 2), which was absent from the whole-cell protein lysate alone (Figure 4(Cb), Lane 1) when probed with the anti-His antibody.

To further confirm Rv3034c-MR interaction, a pull-down assay was performed using Ni-NTA beads. As hypothesized, Ni-NTA beads pulled-down the MR-Rv3034c–His complex, while MR protein alone did not bind to Ni-NTA beads (Figure 4D). A band of 190-kDa was produced for the whole-cell protein lysate flow-through (FT) sample when probed with anti-MR antibody, suggesting this 190-kDa band contained MR. The eluted fractions of the whole-cell protein lysate sample that had been incubated with Rv3034c protein (Figure 4D, right lanes E2, E3) showed a prominent band at ~220-kDa, corresponding to the MR–Rv3034c complex. This ~220-kDa band was observed for the eluted fractions when probed with both anti-MR and anti-His antibodies, further confirming the interaction between Rv3034c and MR.

To test if this interaction of Rv3034c–MR has any role in macrophage response to mycobacteria, we compared the survival of *Rv3034c*-expressing and -depleted *Mtb* strains in the presence or absence of an anti-MR antibody at 24 h p.i. No significant differences in the survival of *Mtb* H37Rv (−ppt^r^) and *Mtb::Rv3034c* (−ppt^r^) were observed in anti-MR-treated and untreated macrophages (Figure 4E). However, the survival of *Mtb* H37Rv (+ppt^r^) and *Mtb::Rv3034c* (+ppt^r^) was significantly reduced (*p* ≤ 0.001, Figure 4E) in anti-MR-treated cells compared with untreated cells, indicating that *Mtb Rv3034c* interacts with MR promoting intracellular persistence.

Next, we determined the levels of peroxisome biogenesis proteins in the presence or absence of MR-antibody. Western blot analysis demonstrated a marked reduction in abundance of the peroxisomal proteins PEX5p, PEX11β, PEX19p, and ABCD3 in anti-MR-blocked and *Mtb*::Rv3034c-(+ppt^r^)-infected macrophages compared with *Mtb*- and *Mtb*::Rv3034c-(−ppt^r^)-infected cells in the absence of anti-MR (Figure 4F). Similarly, pre-treatment with an anti-MR blocking antibody reduced the expression of PEX5p, PEX11β, and ABCD3 in recombinant *Msm*-Rv3034c-infected macrophages (Figure S4A).

Finally, to confirm that MR is prominently involved in the modulation of peroxisomal proteins by *Mtb Rv3034c*, we determined the abundances of PEX11β and ABCD3 in TLR1- (Figure S4B) and TLR2- (Figure S4C) silenced cells. Interestingly, silencing of TLR1 (Figure S4D) and TLR2 (Figure S4E) had no effect on the expression of PEX11β and ABCD3 in *Msm*-*Rv3034c*- and *Msm*-*pSMT3*-infected cells. These results demonstrate that the *Mtb* Rv3034c–MR interaction modulates the expression of peroxisomal proteins during *Mtb* infection. Taken together, these results suggest that *Mtb* Rv3034c interacts with MR on macrophage surfaces to modulate peroxisome functions and alter the redox state within these cells to favor pathogen survival.

## 3. Discussion

Several *Mtb* membrane proteins are known to restrict host immune responses to facilitate bacterial survival. One mechanism is the inhibition of the synthesis of oxidative stress molecules in host cells; however, the underlying molecular mechanisms are not well studied. Here, we report a novel mechanism by which a previously uncharacterized *Mtb* acetyltransferase impedes oxidative stress through the induction of peroxisomes in macrophages. The role of peroxisomes in plants and fungi is well studied; however, their role in mammalian cell during bacterial infections is not well understood [30,31].

Previously, we identified a putative glycoprotein from purified *Mtb* cell walls and proved its role in mycobacterial persistence in macrophages and zebrafish [21]. In this study, we identified another 33-kDa protein, Rv3034c, using ConA lectin chromatography. Extensive in silico and multiple sequence alignment analyses predicted *Rv3034c* to be an acetyltransferase. Several acetyltransferases have been reported in the mycobacterium genome [32]. Purified Rv3034c protein showed significantly higher acetyltransferase activity than BSA, and the enzymatic activity was lost upon heat inactivation, indicating that *Rv3034c* encodes an acetyltransferase. *Mtb* genome analysis also predicted *Rv3034c* to be an essential gene, important for cellular functioning and mycobacterial viability [33,34]. Therefore, we employed two approaches to elucidate its functions. Firstly, we constructed the *Mtb Rv3034c* conditional mutant under the control of the inducible pristinamycin promoter. The absence of pristinamycin resulted in the depletion of *Rv3034c (Mtb*::Rv3034c[−ppt^r^]), whereas the addition of pristinamycin induced the expression of *Rv3034c* (*Mtb*::Rv3034c[+ppt^r^]). In the second approach, we expressed *Rv3034c* in a surrogate *Msm* model (*Msm* Rv3034c). We observed significantly less acetyltransferase activity in *Mtb*::Rv3034c (−ppt^r^) mutant compared with *Msm Rv3034c* and *Mtb*::Rv3034c (+ppt^r^) strains, further suggesting that *Mtb Rv3034c* encodes for an acetyltransferase.

Peroxisomes are known to alter the innate immune responses to fight against infections [35]. Our in vivo and ex vivo results demonstrated that *Mtb Rv3034c* is able to induce peroxisomal protein abundance in infected mice lung tissues and BMDM. Moreover, infection with *Msm Rv3034c* also showed higher expression of peroxisomal biogenesis and transporter proteins in mouse macrophages. This is in line with our previous report that *Mtb Rv3034c* induces peroxisome homeostasis in mouse macrophages.

As Rv3034c is a cell-wall-associated protein, it is presumed that Rv3034c can mediate host–pathogen interactions. Moreover, *Mtb*::Rv3034c(+ppt^r^) strain exhibited an increase in intramacrophage survival, indicating that Rv3034c is also involved in mycobacterial survival in macrophages. *Mtb* acetyltransferase, Eis protein, and arylamine N-acetyltransferase have been shown to be involved in processes promoting *Mtb* survival in macrophages [36,37].

Pathogenic mycobacteria inhibit oxidative burst mechanisms or employ various detoxification pathways to combat oxidative stress. ROS, especially H_2_O_2_, exhibits a strong toxicity effect on invading pathogens [38,39]. Cells utilize several antioxidant enzymes such as superoxide dismutase and catalase to metabolize ROS intermediates. How *Mtb* counteracts host oxidative stress remains elusive. Here, we have provided evidence that *Mtb Rv3034c* is responsible for the inhibition of ROS production in macrophages. This agrees with a previous report where deletion of *Mtb eis* resulted in increased ROS production [32].

ROS metabolism has always been linked to mitochondria [39]; however, peroxisomes also play a crucial role in redox signaling, β-fatty acid oxidation, and lipid homeostasis [8,40]. We observed *Rv3034c* counters ROS production through induction of peroxisome biogenesis during the infection process. Peroxisomes contain different oxidase enzymes such as catalase and glutathione peroxidase [40,41]. We showed that *Mtb* Rv3034c induced the expression of peroxisomal catalase, while the absence of *Rv3034c* reduced the expression of peroxisomal catalase, indicating that Rv3034c can detoxify oxidative radicals through upregulation of catalase enzymes. This corroborates a previous report that *Mtb* possesses SOD and catalase that degrade ROS to combat oxidative stress and helps the pathogen to survive and replicate in macrophages [15].

Limited studies have shown that pathogens such as *Cryptococcus* can induce peroxisomal β-fatty acid oxidation and lipid degradation [42]. These intracellular fungal pathogens were able to use host lipids to aid their intracellular proliferation. Peroxisomes can use long-chain fatty acids to generate energy via the β-fatty acid oxidation pathway. This process requires an import of fatty acids via the peroxisomal membrane-associated ABCD3 transporter [43,44]. ACOX1, ACAA1, and MFP2 are the hallmark enzymes of β-fatty acid oxidation. *Mtb* infection enhanced expression of ACOX1, ACAA1, and MFP2. In contrast to *Mtb Rv3034c* expressing bacteria, the *Mtb Rv3034c* depleted mutant significantly downregulated the expression of these enzymes, indicating that *Rv3034c* induces β-fatty acid oxidation intermediates. Further, presence of *Mtb Rv3034c* induced the expression of the glyoxylate cycle hallmark enzymes such as Icl-1 and MAS in the mycobacteria, suggesting that the end products of β-fatty acid oxidation pathway (acetyl-CoA and short-chain fatty acids) can be metabolized by *Mtb* as nutritional source through the glyoxylate pathway and therefore, can contribute to mycobacterial growth inside host cells [45]. Furthermore, inhibition of peroxisomes by a selective peroxisomal inhibitor, TZ, in infected macrophages increased ROS production, abrogated *Rv3034c*-mediated induction of β-fatty acid oxidation mediators, and decreased *Msm*-*Rv3034c*-induced peroxisomal protein expression. These results suggest that Rv3034c-mediated peroxisome induction is responsible for the observed phenotypes. However, further investigation is required into the regulation of the mycobacterial glyoxylate pathway through modulation of host peroxisomal machinery.

**Figure 5:**
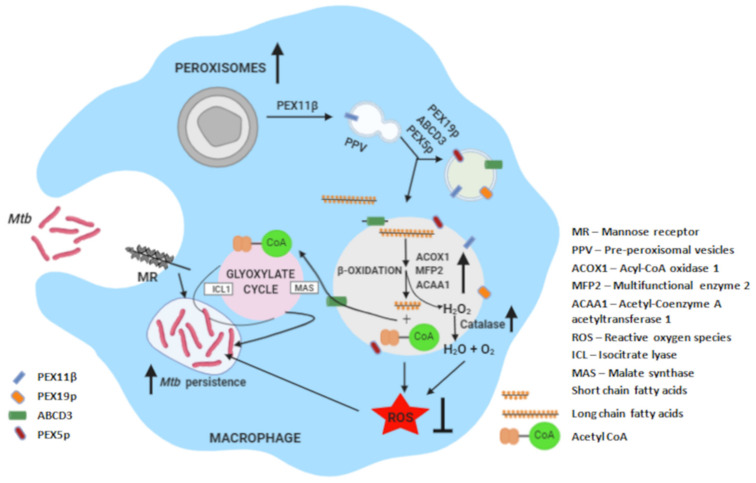
Diagrammatic representation of the role of *Rv3034c* in modulating host immune responses by inhibiting ROS production through induction of peroxisomal proteins and increased bacterial persistence by induction of glyoxylate pathway. Here, we studied that Mtb Rv3034c interacts with the mannose receptor present on the host macrophages. This leads to transcriptional activation of peroxisomal proteins (PEX5p, PEX11β, PEX19p) and related enzymes (such as ACOX1 and ACAA1), as well as expression of antioxidative enzyme catalase. It is considered that ROS generated during peroxisomal β-oxidation is counterbalanced by the overexpression of catalase during *Mtb*Rv3034c infection. The growth and proliferation of pre-peroxisomal vesicles are mediated via PEX11β and PEX19p expression, which further recruits ABCD3. The final maturation occurs via the expression of PEX5p and other peroxins. Peroxisomal β-fatty acid oxidation pathway helps in the metabolism of long-chain fatty acids into short-chain fatty acids and acetylCoA. Induction of peroxisomes post-infection also activates the glyoxylate pathway. Thus, *Mtb*-Rv3034c-mediated events help in promoting persistence of *Mtb* in macrophages. The diagram was created with BioRender.com.

Induction of peroxisome biogenesis was dependent on the interaction between *Rv3034c* and MR. Purified *Mtb Rv3034c* showed strong glycoprotein staining and specificity toward mannose-specific ConA lectin. Following anti-MR blocking, peroxisome biogenesis and peroxisome-associated proteins were downregulated. Pull-down assays revealed that *Rv3034c* and MR can directly interact in vitro. In contrast, silencing of TLR1 and TLR2 did not affect peroxisomal induction by *Rv3034c*. These results strongly suggest that Rv3034c is a mannosylated glycoprotein that interacts with MR with a subsequent modulatory effect on peroxisome biogenesis. This is in agreement with a previous report showing that an *Mtb*-mannosylated glycolipo protein can attach to MRs present on the surface of macrophages [29]. Subcellular localization studies using membrane-specific dye showed that *Rv3034c* is predominantly localized in the bacterial membrane. Strong membrane localization was also observed in the GFP-tagged *Rv3034c* C-terminus, which encodes an acetyltransferase domain. These results demonstrate that *Mtb Rv3034c* is a membrane-associated protein. Infection assays showed that the survival of *Mtb*-*Rv3034c*-expressing strains was significantly downregulated in anti-MR-treated but not untreated cells. In conclusion, we identified a novel *Mtb* acetyltransferase and revealed previously unknown mechanistic insights into how *Rv3034c* interferes with host immunity and promotes generation of a suitable niche for intracellular bacillary persistence by modulating oxidative stress responses through induction of peroxisomes to establish a successful infection (Figure 5), and thus can be considered a potential antimycobacterial drug target. More importantly, peroxisomes were revealed as putative target organelles to modulate innate immune responses as host-directed therapies accompanying antibiotic treatment.

## 4. Materials and Methods

### 4.1. Bacterial Strains, Cell Lines and Reagents

*Mycobacterium tuberculosis* H37Rv and *Mycobacterium smegmatis* mc^2^155 were grown in Middlebrook’s 7H9 broth medium (Difco, Franklin Lakes, NJ, USA) containing 0.05% Tween 80 at 37 °C at 120 r.p.m. *Escherichia coli* XL-10 Gold (Stratagene, San Diego, CA, USA) was grown in Luria–Bertani (LB) broth supplemented with 20 µg/mL tetracycline (Sigma Aldrich, St. Louis, MO, USA). Mouse macrophage RAW264.7 [46] and human monocytic THP-1 cells [47] were maintained in DMEM and RPMI medium (Gibco Invitrogen, Waltham, MA, USA) as described previously [21,48]. Mouse bone-marrow-derived macrophages were cultured in DMEM and supplemented with 10% FCS and 2% LCSN. DCFH and thioridazine hydrochloride were procured from Sigma (St. Louis, MO, USA). Protease inhibitor cocktail was purchased from Roche (Basel, Switzerland). TRIzol and DHE were obtained from Invitrogen (Waltham, MA, USA). The cDNA synthesis kit was procured from Fermentas (Waltham, MA, USA). Pristinamycin was procured from Molcan Corporation (Richmond Hill, ON, Canada). PEX5p, PEX11β, PEX19p, and ABCD3 antibodies were procured from Abcam (Cambridge, UK). His Tag (# 2365s), Mannose receptor (#1281S), GAPDH, and β-actin antibodies were obtained from Cell Signaling technologies (Danvers, Massachusetts, USA). Anti-MR (CD206) antibody was procured from eBiosciences (San Diego, CA, USA). TLR-1/2 siRNAs were obtained from Santa Cruz Biotechnology (Dallas, TX, USA). FM4-64 FX dye (Catalog # F34653), Anti-GFP (Catalog #MA5-15256), and goat anti-mouse secondary (Catalog #A32723) antibodies were purchased from Thermo Scientific, Waltham, MA, USA.

### 4.2. Ethical Statement

Studies involving virulent mycobacterial strains were carried out at the Biosafety level 3 facility at the National Institute of Immunology, New Delhi; Leibniz Lung Centre, Borstel, Germany; and Albany Medical College, Albany, NY, USA.

### 4.3. ConA Affinity Purification, Lectin Hybridization, and Mass Spectrometry Analysis

Purified *Mtb* cell walls were delipidated, extracted using methanol and chloroform, and then subjected to ConA affinity purification as described previously [21]. Briefly, the delipidated cell walls were resuspended in lectin buffer (200 µL) and added to 100 µL of equilibrated ConA-agarose conjugated beads in a 1:1 ratio. Proteins were allowed to bind to mannose-specific residues of ConA-lectin for 4–6 h at room temperature. Bound proteins were then eluted by the addition of 50 µL of 0.4 mM competitive sugar solution. Samples were separated on 10% SDS-PAGE gels. Protein bands were identified by LC-ESI mass spectrometry analysis as described before [21].

### 4.4. Construction of Mtb Rv3034c Conditional Mutant

The *Rv3034c* mutant was generated using pristinamycin (ppt^r^) based suicidal vector containing pfurA-pip promoter-operator system, oriE, and hygromycin as an antibiotic selection marker [49]. The 5′ region of *Rv3034c* (−20 to 600 bp) was amplified from the *Mtb*H37Rv genomic DNA. Amplicons were cloned into *Nco1* and *Sph1* sites of suicide vector to generate pAZ-3034 construct and positive transformants were selected on 7H10 agar containing hygromycin (100 µg/mL) and pristinamycin (2 µg/mL) as described previously [16]. *Mtb*H37Rv wild-type and *Mtb*::Rv3034c conditional mutant grown in the presence and absence of pristinamycin were designated as “+ppt^r^ and −ppt^r^, respectively.

### 4.5. Cloning and Expression of Rv3034c in Msm

*Mtb Rv3034c*, N terminus-*Rv3034c* (1–62 bp), and C-terminus *Rv3034c* (63–903 bp) were PCR amplified using gene-specific primers (Table 1) and *Mtb* genomic DNA as a template. The PCR products were cloned into pSMT3 shuttle vector and the positive transformants were selected as described previously [21]. Similarly, Msm-pSC300-GFP strain was constructed by cloning *Mtb Rv3034c* into pSC300 vector and the positive transformants were selected as described previously [48]. *Msm* harbouring empty pSC300 and GFP-fusion proteins were designated as Msm-pSC300 and Msm-pSC300:Rv3034c, respectively. Similarly, Msm-pSC300:Rv3034c-C strain was generated that expressed C-terminus acetyltransferase domain of *Rv3034c* using gene specific primers.

### 4.6. Rv3034c Protein Purification

The full-length *Rv3034c* gene was amplified from *Mtb* H37Rv genome, digested with *Nde*I and *Hind*III, and cloned into pET21b vector. The recombinant plasmid was transformed into *E. coli* DH5α. Positive clones were confirmed by colony PCR and sequencing using gene-specific primers. Finally, Rv3034c protein was expressed in *E. coli* BL21 (DE3). Protein expression was induced at 16 °C with 0.4–0.6 mM IPTG (isopropyl-b-D-thiogalactopyranoside, Sigma Aldrich, St. Louis, MO, USA). Cells were harvested and lysed using lysis buffer (20 mM Tris/HCl, pH 8.0, 5% glycerol 300 mMNaCl, and lysozyme 2 mg/mL) containing protease inhibitor cocktail (Roche, Basel, Switzerland). Harvested cells were then sonicated, centrifuged at 10,000× *g*. Rv3034c-His-tagged protein was purified by affinity chromatography using Ni-NTA resin (Qiagen, Hilden, Germany).

### 4.7. Cellular Localization of Rv3034c and GFP Immunostaining

GFP immunostaining of Msm-pSC300, Msm-pSC300:Rv3034c, and Msm-pSC300:Rv3034c-C strains were performed as described previously [48]. Briefly, bacterial pellets were stained with a lipophilic probe FM 4-64FX [*N*-(3-triethylammoniumpropyl)-4-(6-(4-(diethylamino) phenyl) hexatrienyl) pyridinium dibromide] (Thermo Scientific, MA, USA) at a concentration of 5 µg/mL for 10 min. Cells were fixed with 2.8% formaldehyde containing 0.04% glutaraldehyde for 15 min at room temperature (RT). Then cells were permeabilized with 0.1% triton X-100 in 1X PBS for 45 min. Cells were incubated in PBS containing 100 µg of lysozyme per ml and 5 mM EDTA for 45 min at RT, washed, and immuno-stained with anti-GFP antibody (MA5-15256, Thermo Scientific) for 1h at RT, goat antimouse secondary antibody, and Alexa Fluor 488 (Thermo Scientific, A32723) for 30 min. The cells were analyzed under fluorescence microscope (Olympus, Tokyo, Japan) and the images were analyzed using the ImageJ software version 1.53n (NIH).

### 4.8. Acetyltransferase Assay

Mycobacterial strains were grown as described above. The standard assay solution containing the initiation mixture (50 mM Tris HCl pH 7.5, 5 mM MgCl_2_, 0.4 mM Acetyl CoA, 9.5 mM NTB (2-nitro-5-thiobenzoate), protein lysate, and termination mixture (50 mM Tris HCl pH 7.5 and 6 M guanidine hydrochloride along with 0.2 mM DTNB and 1 mM EDTA) were incubated for 10 min and the absorbance was measured at 412 nm for 10 min [50]. Absorbance at 0 min is the absorbance measured immediately after 10 min of incubation. Absorbance values were calculated after deduction from negative control samples (which contained buffer solution except the protein).

### 4.9. Intracellular Survival Assay

*Mtb* H37Rv (±ppt^r^) and *Mtb*::Rv3034c (±ppt^r^) were grown in the presence of pristinamycin in 7H9 media for 72 h. Peritoneal macrophages (5 × 10^5^ cells/well) were seeded and infected at an MOI of 1:5 with *Mtb* H37Rv (±ppt^r^) and *Mtb*::Rv3034c (±ppt^r^) in presence and absence of pristinamycin. Cells were lysed after 24, 48 and 72 h, and CFU were enumerated after 21 days.

### 4.10. Isolation of Mouse Peritoneal Macrophages

Peritoneal macrophages were isolated from 6–8-week-old female BALB/c mice as described previously [21]. Briefly, mice were euthanized and peritoneal fluid was removed and centrifuged at 400× *g* for 10 min at 4 °C. The cell pellet was resuspended in DMEM F-12 medium and cells were seeded onto six-well plates. Approximately, 2 × 10^6^ cells were seeded onto six-well plates and incubated overnight, after which the nonadherent cells were washed off with DMEM-antibiotic medium. Adherent cells were then cultured and used for infection as described [21].

### 4.11. Flow Cytometry Analysis

RAW264.7 (3 × 10^6^ cells/well) were seeded onto six-well plates and infected with *Msm* pSMT3 and *Msm*Rv3034c at an MOI 10 for 24 h as described above. The generation of total ROS was measured by using 2′, 7′-dichlorofluorescein diacetate (DCFH-DA) dye using flow cytometer (BD FACS Fortessa, NJ, USA) and the FACS Diva software as described previously [21]. Forward scatter (FSC) area vs. height measurement was done to remove clumps for single cell analysis and single cells falling along a diagonal were chosen for further analysis. Forward scatter (FSC) and side scatter (SSC) were used to gate viable and single cell events. Appropriate gating was done to exclude debris and dead cells from analysis by categorizing low-FSC events as debris, and low FSC and high SSC as dead cells. A compact cell population was thus gated based on size and granularity. Gated cells were further analyzed for uptake of impermeable propidium iodide (PI) (Invitrogen, MA, USA) stain to determine live versus dead cells. Gated FITC positive cells from stained and unstained controls, *Msm* pSMT3, and *Msm* Rv3034c were overlayed to determine shift in the population using FlowJo (Ashland, OR, USA).

### 4.12. Mouse Infection

The wild-type strain of *M. tuberculosis* (*Mtb*) H37Rv was used in these studies. Bacteria were cultured in 7H9 medium containing 0.05% Tween 80 and OADC enrichment (Becton Dickinson, Franklin Lakes, NJ, USA). For infections, mycobacteria were suspended in phosphate-buffered saline (PBS)-Tween 80 (0.05%); clumps were dissociated by sonication and ~200 CFU were delivered via the respiratory route using an aerosol generation device (Glas-Col, Terre Haute, IN, USA). Eight-week-old male C57BL/6 (stock no. 000,664 from Jackson Lab, Bar Harbor, ME, USA) were infected with ~200 CFU of aerosolized *Mtb*H37Rv. Six weeks post-infection, mice were euthanized and lungs were harvested, fixed in 10% buffered formaldehyde, and processed for immunofluorescence microscopy as follows.

### 4.13. Immunofluorescence Microscopy

Paraffin-embeded lung tissue sections were cut at 5 μm thickness, mounted on ultraclean glass slides covered in silane, deparaffinized, then dehydrated and rehydrated using the following steps: Ethanol solutions (30, 50, 70, 90, 95, and 100% for 3 min each), xylenes (two different solutions for 10 min each), and ethanol solutions (100, 95, 90, 70, 50, and 30 for 3 min each). The slides were washed once in Tris buffer saline (TBS) for 5 min. Slices were subjected to antigen retrieval by boiling in sodium citrate buffer at pH = 6.0 for 20 min and incubated in 0.1% Triton-X 100 for 5 min. Slices were removed and allowed to equilibrate to room temperature for at least 20 min and rinsed with distilled water. Tissue sections were blocked (blocking solution; 0.5 M EDTA, 1% BSA, in PBS) and incubated overnight in primary antibodies against the proteins related to our studies. Sections were stained for nuclei (DAPI, blue staining), antimouse ABCD3 (cat.no. NBP1-97258, Novus Bio, Englewood, CO, USA) or antimouse PEX14p (cat no. NBP2-33556, Novus Bio) to identify Peroxisomes (Cy3 red staining). As controls, preimmune serum and isotype matched controls were used. After incubation, the tissues were washed several times with sterile TBS at room temperature and incubated in the respective secondary antibodies (antirabbit conjugated to conjugated to Cy3) for at least 2h at room temperature. Tissue sections were mounted using Prolong Gold Antifade reagent (Invitrogen, Grand Island, NY, USA) with DAPI, and the tissue sections were examined using an ECHO Revolve 4 microscope. Isotype matched control antibodies were used for checking antibody specificity.

RAW264.7 macrophages were seeded on glass coverslips in six-well tissue culture plates and infected with *Msm* pSMT3 and *Msm*Rv3034c as described above. Cells were fixed and permeabilized as described previously [48]. Cells were then stained with PEX5p, and ABCD3 (Abcam, Cambridge, UK) for 2 h at RT followed by fluorophore-conjugated secondary antibody. The stained cells were observed under fluorescence microscope (Olympus, Tokyo, Japan) and the images were analyzed using the ImageJ software version 1.53n (NIH).

Macrophages were infected with *Mtb* H37Rv and stained with PEX14p, PEX19p, ACOX1, MFP2, ACAA1, ABCD3, and CAT. The slides were analyzed using a Nikon confocal laser scanning microscope (Nikon EclispseTi inverted microscope, Tokyo, Japan). Images were captured with a 63× objective, 1× zoom and 12 times sampling. All images were processed with Adobe Photoshop CS5 (Version 12, San Jose, CA, USA).

### 4.14. siRNA Transfection

For TLR1/2 silencing, siRNA duplexes against TLR-1 and 2 were designed and synthesized at a 10 nmol concentration. Silencing was performed in THP-1 cells as described previously [48]. Cells were then infected with *Msm* pSMT3 and *Msm* Rv3034c as described above. Silencing efficiency was determined by RT-PCR using gene-specific primers.

### 4.15. Quantitative Real-Time PCR Analysis

To check the intracellular expression of *Rv3034c*, *Mtb*, and *Mtb*::Rv3034c, mutants were grown in 7H9-OADC medium supplemented with hygromycin (100 µg/mL) and pristinamycin (2µg/mL) (+ppt^r^). For depletion of *Rv3034c*, *Mtb* H37Rv, and *Mtb*::Rv3034c, mutants were grown in the absence of pristinamycin (−ppt^r^). Peritoneal macrophages were seeded, infected, and lysed as described above. Total RNA was isolated followed by cDNA synthesis, and qRT-PCR amplification was performed using gene-specific primers in a Real Plex master cycler (Eppendorf, Hamburg, Germany) with initial denaturation at 95 °C for 10 min, final denaturation at 95 °C for 30 sec, annealing at 52 °C for 30 s, and extension at 72 °C for 30 s to generate 200 bp amplicons. Similarly, expression of peroxisomal *catalase, Acox1, Acaa1, Mfp2, Icl1, Mas* were quantified by isolating total RNA from uninfected and infected macrophages using gene specific primers (Table 3). mRNA levels were normalized to transcript levels of *sigA* and GAPDH, respectively, and relative fold changes were calculated.

### 4.16. Western Blot Analysis

RAW264.7 and THP-1 (10^6^) cells were seeded in six-well or 60 mm tissue culture plates. Cells were infected with mycobacterial strains in the presence and absence of MR-antibody blocker (1:1000) 12 h prior to infection. Cells were harvested at the indicated time points and protein samples were prepared by cell lysis using 1X SDS sample buffer (62.5 mM Tris-HCl, pH 6.8, 2% *w*/*v* SDS, 10% glycerol, 50 mMDTT, 0.15 *w*/*v* bromophenol blue) supplemented with a protease inhibitor cocktail. Proteins were electrophoresed in 12% SDS-PAGE, transferred to a PVDF membrane, and then membranes were incubated with primary rabbit IgG antibodies (dilution 1: 5000) overnight at 4 °C and secondary goat antirabbit antibodies (1: 5000) for 2 h. The relative band densities were quantified relative to respective loading controls using the ImageJ software (National Institutes of Health, Bethesda, MD, USA). Protein–protein interaction was also studied by using the Western blot technique [51]. Whole-cell protein lysates were isolated from MR-expressing mouse macrophages and used as the prey protein.

### 4.17. Pull-Down Assay

Whole-cell protein lysates isolated from RAW 264.7 cells and purified Rv3034c-His protein were added to Ni-NTA beads either alone or in combination for 2 h on a rocker at 4 °C. The flow-through (FT) samples were first collected and the beads were then washed (W) with ice-cold equilibration buffer (20 mM Tris/HCl, pH 8.0, 5% glycerol 300 mM NaCl) containing 20 mM imidazole. Finally, bound proteins were eluted (E1-3) in buffer containing 300 mM imidazole. Fractions were electrophoresed on 12% SDS-PAGE. Western blotting was performed with the anti-MR antibody [52].

### 4.18. Statistical Analysis

Graphs were generated using GraphPad Prism (Prism 5.0, San Diego, CA, USA). Statistically significant differences between groups were determined by one-way Analysis of Variance (ANOVA) using Dunnett’s Multiple Comparison Tests and two-way ANOVA with Bonferroni post-tests. Significance was referred as * for *p* < 0.05, ** for *p* < 0.01, and *** for *p* ≤ 0.001 and ns for non-significant.

## Figures and Tables

**Table 1:** Oligos used in the construction of *M. tuberculosis* Rv3034c mutants.

Gene Name	Sequence (5′>>3′)
*Rv3034c*-Forward	GGA TCC GTG AAC GTC CTC AGT TTG GGC TCG T
*Rv3034c*-Reverse	AAG CTT CTA GCG GGC CGC CTT CTT GC
*Rv3034c N-ter* Reverse	TCACCGCCCCCGCGGGGGCGGGCCGCA
*Rv3034c C-ter* Forward	AAAACTGCAGGTGCCACCGGCGTCACCTCGC
*Mtb::Rv3034*(±ppt^r^) Forward	CACC CCA TGG GTG TAC GTG GTG TAA GTG TCG
*Mtb::Rv3034*(±ppt^r^) Reverse	AGCT GCA TGC AGCACCAGTCGGCCATTAGCA
*Msm-pSC300:Rv3034c* Forward	ATGGATCCCTGCAGGTGAACGTCCTCAGTTTGGGCT
*Msm-pSC300:Rv3034c* Reverse	ATTAAGCTTGATATC GCGGGCCGCCTTCTTGCGTT
*MsmpSC300:Rv3034cC-ter* Forward	ATTAAGCTT GATATCGAATTCCTGCAGCT

**Table 2:** Mass spectrometry results showing theoretical mass match with experimental mass and predicted peptide sequence for *Rv3034c*.

Start–End	Observed Mass	Mr(expt)	Delta	Sequence
1–16	1648.7180	1647.7107	−0.1296	MNVLSLGSSSGVVWGR.V
17–32	1468.4590	1467.4517	−0.3528	R.VPITAPAGAATGVTSR.A
33–40	915.0550	914.0477	−0.3551	R.ADAHSQMR.R
42–50	936.1210	935.1137	−0.3575	R.YAQTGPTAK.L
51–66	1755.8930	1754.8857	0.0261	K.LSSAPMTTMWGAPLHR.R
81–88	892.5120	891.5047	−0.0382	K.FLTLASLK.W
95–103	1168.1250	1167.1177	−0.4899	R.AYTPWYLVR.Y
104–106	524.2900	523.2827	0.0284	R.YWR.L
112–120	1034.6180	1033.6107	0.0075	K.LANPHIITR.G
121–127	751.4140	750.4067	−0.0031	R.GMVFLGK.G
128–144	1833.0130	1832.0057	0.0265	K.GVEIHATPELAQLEIGR.W
145–151	854.5430	853.5357	0.0911	R.WVHIGDK.N
152–155	503.7740	502.7667	0.4804	K.NTIR.A
156–162	769.5000	768.4927	0.1049	R.AHEGSLR.F
167–171	543.3640	542.3567	0.0027	K.VVLGR.D
221–230	1085.7020	1084.6947	0.1030	R.IGPDTWIGVK.V
231–235	573.3350	572.3277	−0.0369	K.VSVLR.G
236–241	604.3450	603.3377	0.0037	R.GTTIGR.G
242–252	1154.6410	1153.6337	0.0311	R.GCVLGSHAVVR.G
253–267	1429.8940	1428.8867	0.1255	R.GAIPDYSIAVGAPAK.V
284–295	1242.6740	1241.6667	0.0052	R.AELAAALADIER.K

**Table 3:** Oligos used in qRT-PCR analysis.

Gene Name	Sequence (5′>>3′)
*Rv3034c*-Forward	ATT CTC AGA TGC GCC GAT AC
*Rv3034c*-Reverse	AGT AGC GCA CCA GGT ACC AC
*Mfp2*-Forward	GCATTGATGTGGTGGTGAAC
*Mfp2*-Reverse	GAATGCGGCCATAGTTCTGT
*Acaa1*-Forward	GGCCTTCTTTCAAGGGAAAC
*Acaa1*-Reverse	CTAAGCCCTGACGACGAGAC
*Catalase*-Forward	ACATGGTCTGGGACTTCTGG
*Catalase*-Reverse	CAAGTTTTTGATGCCCTGGT
*Acox1*-Forward	GCTGAGGAACCTGTGTCTCT
*Acox1*-Reverse	TCAAAGGCATCCACCAAAGC
*Icl1*-Forward	GTTTAGCGAAGCGGTGAAAG
*Icl1*-Reverse	CCGCCAGGGTAATAAACTGA
*Mas*-Forward	GCACCGGCAGCATTTATATT
*Mas*-Reverse	GATCCAGAAAGCCGGTGTTA
*SigA*-Forward	CCAAGGGCTACAAGTTCTCG
*SigA*-Reverse	TGGATCTCCAGCACCTTCTC
*GAPDH*-Forward	AGGGCCCTGACAACTCTTTT
*GAPDH*-Reverse	AGGGGCTACATGGCAACTG

## Data Availability

The study did not report any data.

## Supplementary Materials

The following are available online at https://www.mdpi.com/article/10.3390/ijms23052584/s1.
