# Supplementary material for: Mycobacterium tuberculosis Acetyltransferase Suppresses Oxidative Stress by Inducing Peroxisome Formation in Macrophages
Source: Int J Mol Sci. 2022 Feb 26;23(5):2584. doi: 10.3390/ijms23052584 (PMC8909987; doi:10.3390/ijms23052584)
Supplement: Supplementary file 1 [file ijms-23-02584-s001.zip › ijms-1436843-sm.pdf]

Supplementary Materials

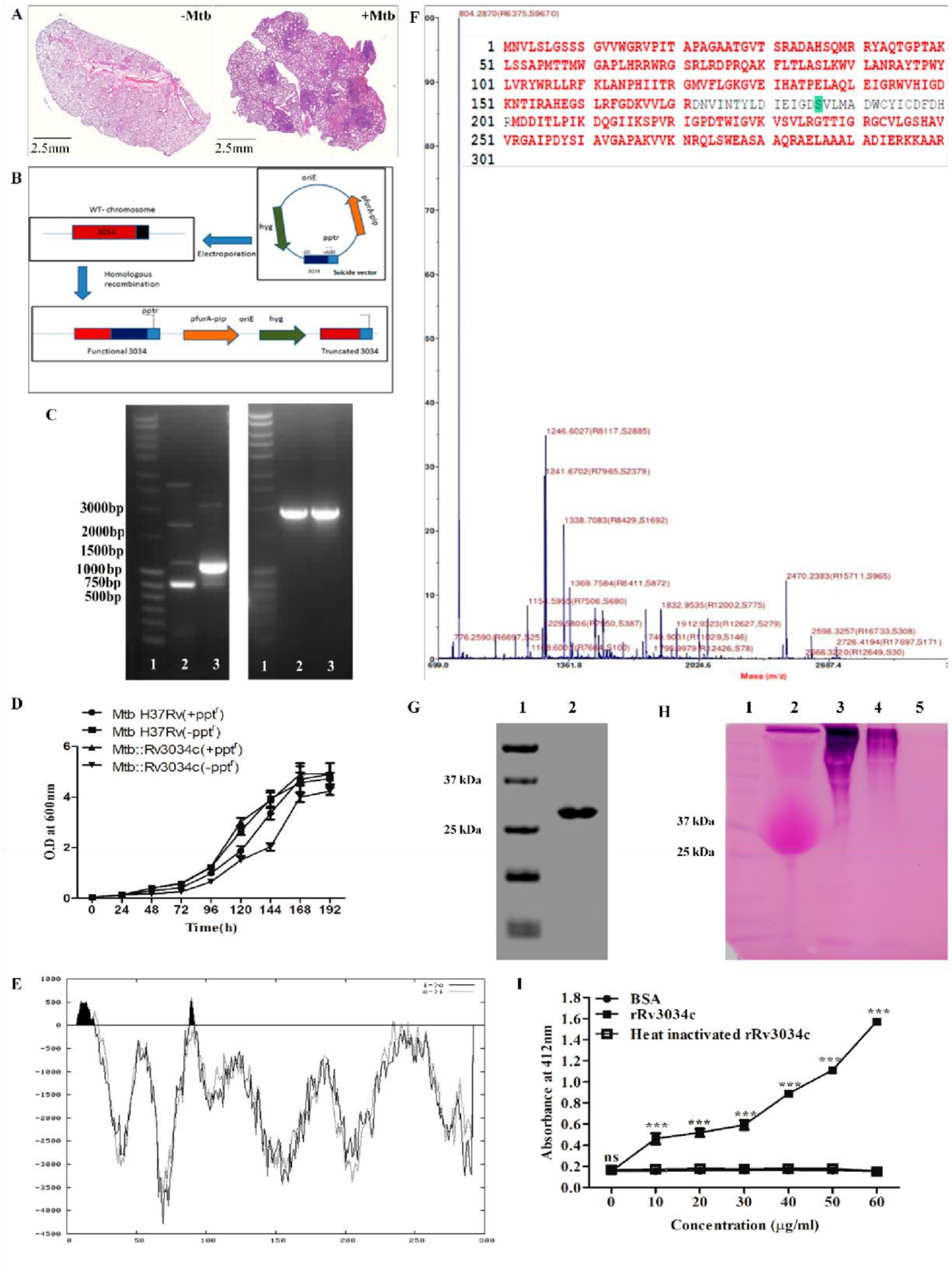

(**I**)

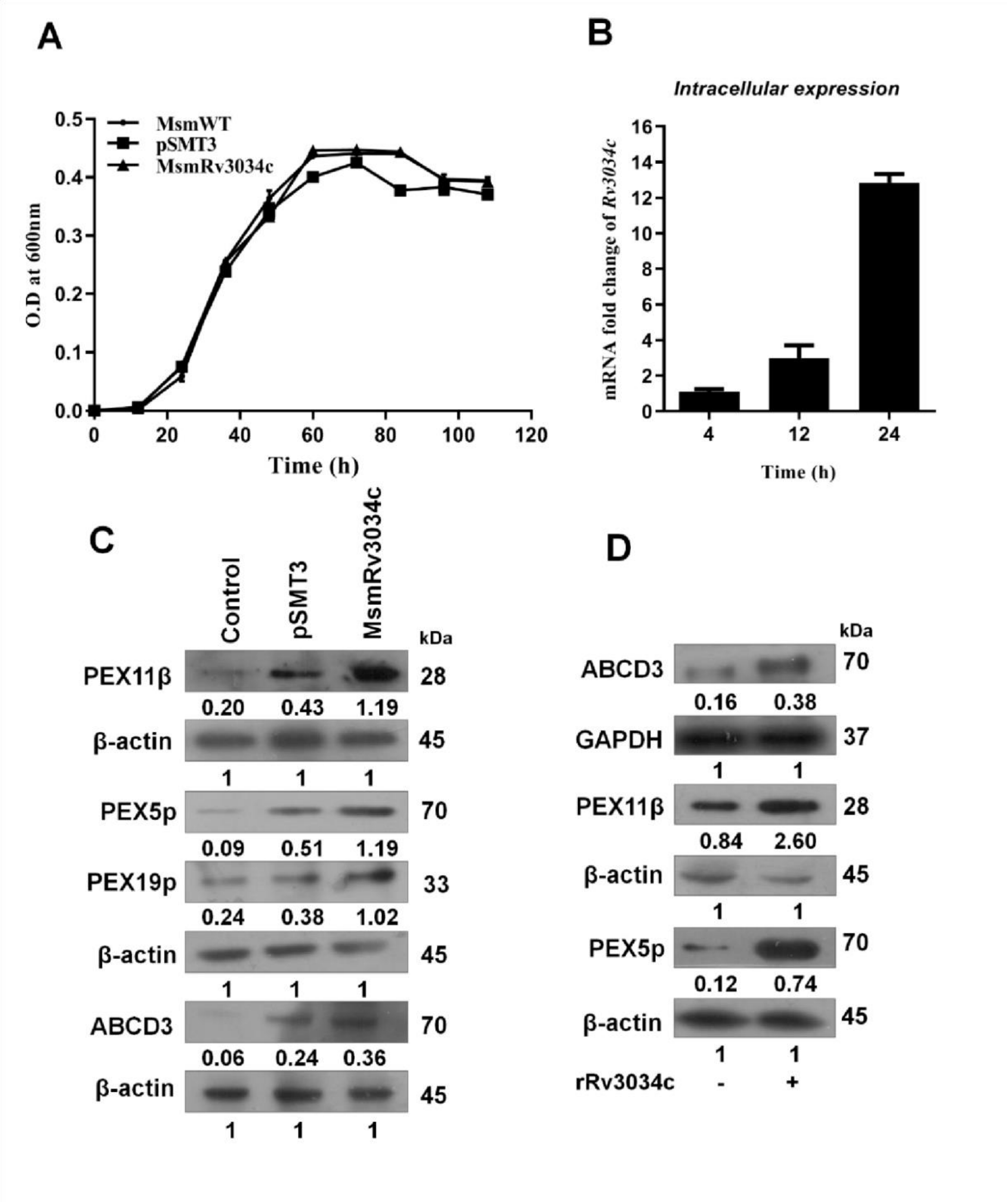

(**II**)

Figure S1. (**I**). Construction and confirmation of *Mtb* Rv3034c conditional mutants and mass spectrometry analysis, in vitro growth kinetics, purification and glycostaining of *Mtb* Rv3034c. (**A**) Hematoxylin and eosin-stained paraffin sections of lung tissues from uninfected and *Mtb*H7Rv infected mice (n = 5, males). Scale bar, 2.5mm. (**B**) Schematic representation of construction of *Mtb Rv3034c* conditional mutant using pristinamycin based suicidal vector. (**C**) PCR products were obtained from *Mtb* H37Rv genomic DNA using different primer pairs. PCR amplified products obtained from *Mtb* H37Rv and putative *Mtb*::Rv3034c mutant (Lane 2) were resolved on 1% agarose gel. PCR reactions in the left side panel were performed using ppt^r promoter forward primer and *Rv3034c* reverse primer. Presence of specific 1.1kb band in Lane 3 (left side panel) is indicative of full length Rv3034c gene placed next to ppt^r promoter. Control PCR reactions were performed (right side of the panel Lane 2 and 3) with *ftsQ* gene forward and reverse primers. Lane 1 in both the gel panels represents the 1kb DNA ladder.

The genomic DNA from wild-type Mtb gave non-specific bands as it lacks the ppt^r promoter region. Control PCR reactions were performed to validate that template was added in both the reactions. (**D**) In-vitro growth pattern of *Mtb* H37Rv (-ppt^r), *Mtb*H37Rv (+ppt^r), *Mtb*::Rv3034c (-ppt^r), *Mtb*::Rv3034c(+ppt^r) strains in 7H9 medium. The O.D. was measured at 600 nm at the indicated time points. (**E**)Transmembrane domain analysis of Rv3034c was performed by TMpred. (**F**) Identification of 33-kDa protein by LC-ESI mass spectrometry. The peptide coverage identified from the mass spectrometry analysis has been highlighted (red) and Serine 186 residue predicted to be the glycosylation site of Rv3034c has been highlighted as well (green). (**G**) Purification of Rv3034c protein using Ni-NTA resin affinity chromatography. Lane 1-pre-stained protein ladder, and lane 2-purified Rv3034c protein. (**H**) Glycoprotein staining using Schiff base stain. Protein ladder (lane 1), purified Rv3034c 33-kDa protein (lane 2), different concentrations of Snail glycoprotein (lane 3 and 4) and soybean trypsin inhibitor (lane 5) proteins were used as positive and negative controls, respectively. (**I**) Acetyltransferase activity was measured at different doses of purified rRv3034c for 5 min. BSA and heat inactivated rRv3034c protein were used as negative controls. The absorbance values plotted were obtained after deduction from the absorbance value of the control sample. Statistical significance was performed with one-way ANOVA. For acetyltransferase activity, statistical significance was performed with two-way ANOVA Bonferroni post tests. Data represent mean ± SD; ns for non-significant and *** for $p \leq 0.001$. (**II**). Determination of in vitro growth kinetics and intracellular expression of *Rv3034c* and expression of peroxisomal proteins in infected mouse macrophages. (**A**) In-vitro growth pattern of *Msm* WT, *Msm* pSMT3 (pSMT3) and recombinant *Msm Rv3034c* strains in 7H9 medium. The O.D. was measured at 600 nm at the indicated time points. (**B**) Expression of *Rv3034c* was determined by qRTPCR. Total RNA was isolated from *Msm Rv3034c* infected RAW264.7 macrophages (intracellular) at 4, 12 and 24 h time points. 4 h time point was used as the calibrator and assigned a value of 1. The expression values were normalized with house-keeping *sigA* gene. (**C**) Expression of PEX5p, PEX11β, PEX19p and ABCD3 were determined in RAW264.7 macrophages infected with *Msm*pSMT3 and *Msm Rv3034c* strains. (**D**) Expression of PEX11β, PEX5p and ABCD3 were determined in purified rRv3034c protein (60μg) treated mouse macrophages. Untreated cells were used as control. Experiments were performed in duplicates.

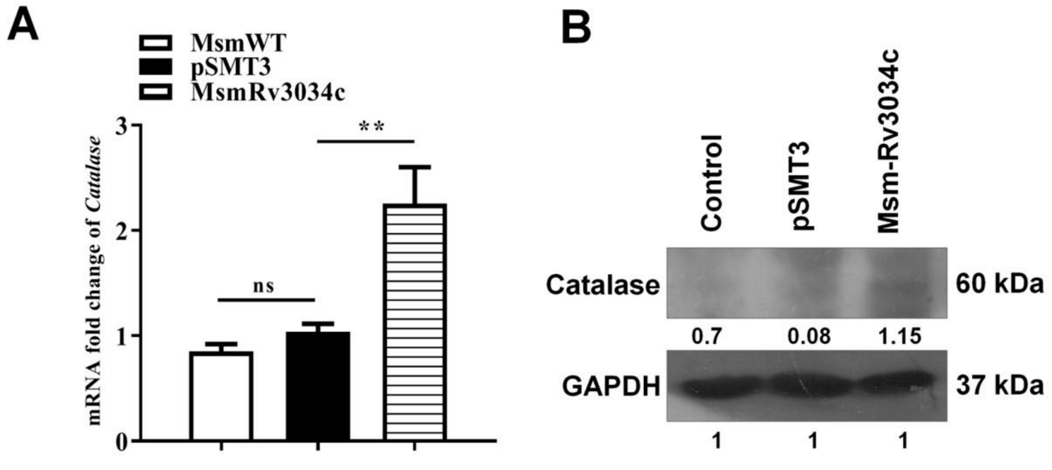

Figure S2. Determination of expression of peroxisomal catalase and SOD. (**A** and **B**) qRT-PCR analysis and western blot analysis of peroxisomal catalase, respectively were performed from (**A**) total RNA isolated from RAW264.7 macrophages infected with *Msm* WT, *Msm* pSMT3 and *Msm Rv3034c* after 24 h. The expression values were normalized with *GAPDH* gene, (**B**) from RAW264.7 macrophages infected with *Msm* pSMT3 and *Msm Rv3034c*. (Experiments were performed in duplicates. Data represent mean ± SD; ** for $p < 0.01$ and ns for non-significant.

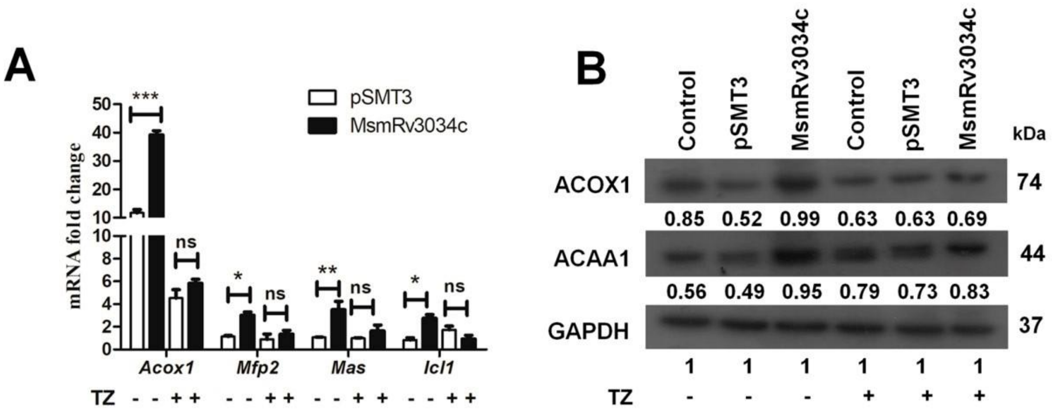

Figure S3. Determination of expression of peroxisomal β-fatty acid oxidation and bacterial glyoxylate cycle intermediates after treatment with peroxisomal β-oxidation inhibitor. (**A**) Transcriptional analysis to determine the expression of peroxisomal β-fatty acid oxidation and glyoxylate genes in *Msm* pSMT3 and *Msm* Rv3034c infected cells with TZ treatment. The expression values were normalized with *GAPDH* and *sigA* gene. (**B**) Western blot analysis was performed to check the expression of ACOX1 and ACAA1 in RAW264.7 macrophages infected with *Msm* pSMT3 and *Msm Rv3034c* in absence and presence of peroxisome inhibitor (TZ) after 24 h infection. Experiments were performed in duplicates. Data represent mean ± SD; * for $p < 0.05$, ** for $p < 0.01$, *** for $p \leq 0.001$ and ns for non-significant.

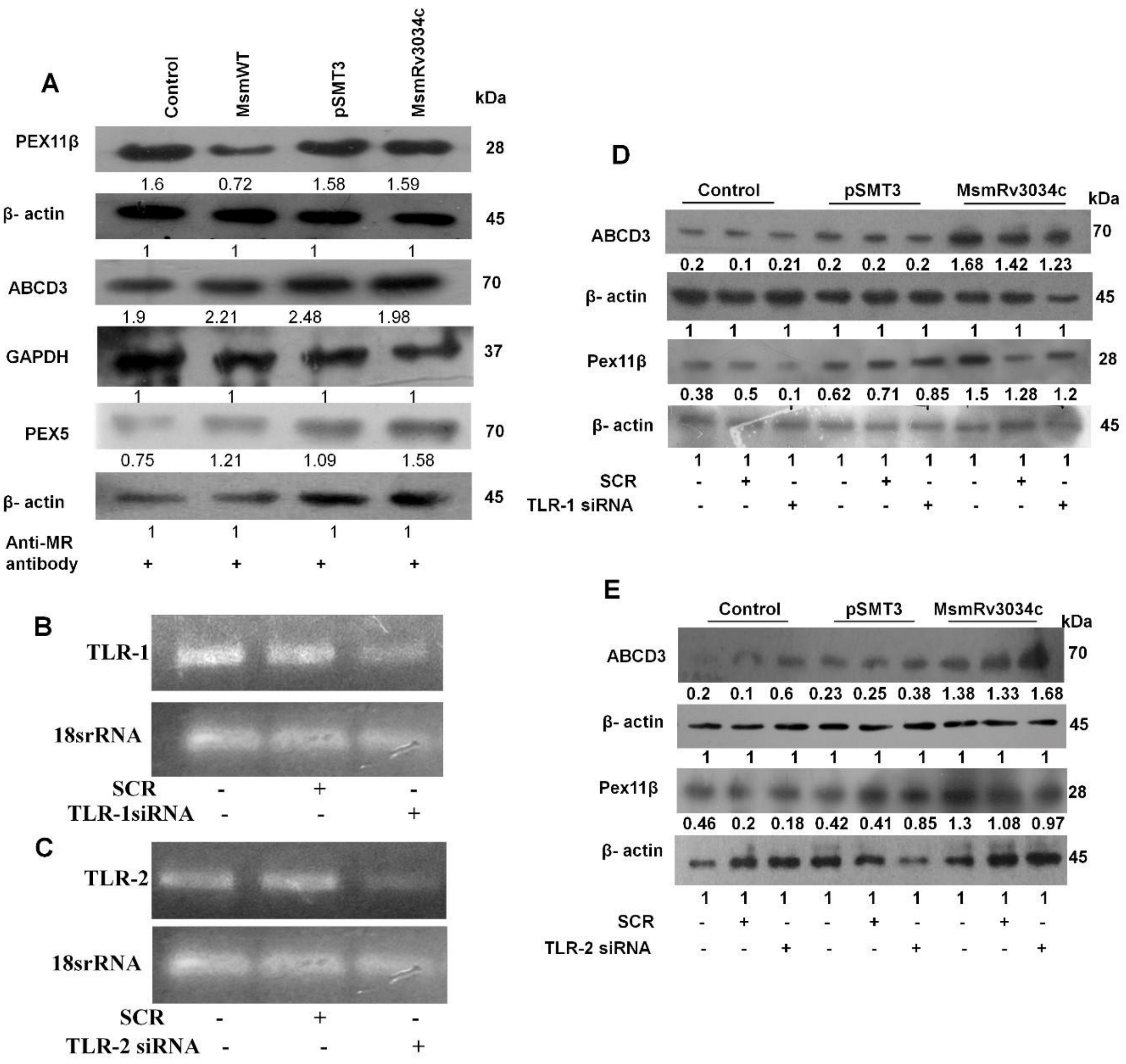

Figure S4. Determination of expression of peroxisomal proteins in presence *Msm* infected mouse macrophages and TLR-1 and TLR-2 silenced and infected macrophages. (**A**) Determination of expression of PEX11β, PEX5p and ABCD3 in presence of MR blocker and *Msm*WT, *Msm*pSMT3 and *Msm Rv3034c* infected macrophages after 24 h. (**B** and **C**) Determination of TLR1 and TLR2 silencing efficiency. (**D** and **E**) Expression of peroxisomal markers (PEX11β and ABCD3) was checked in TLR1 (**D**) and TLR2 (**E**) silenced and *Msm* pSMT3 and *Msm* Rv3034c infected cells. Cells transfected with scrambled siRNA (SCR) were used as control. Experiments were performed in duplicates.
